# Emergency Identification of Endotracheal Tube Tip via Ultrasonography Used by Trained Nurse in the Neonatal Intensive Care Unit (NICU)

**DOI:** 10.3390/diagnostics15030262

**Published:** 2025-01-23

**Authors:** Athanasia Voulgaridou, Savas Deftereos, Pelagia Chloropoulou, Konstantina Bekiaridou, Emmanouela Tsouvala, Rozita Meziridou, Soultana Foutzitzi, Christos Kaselas, Xenophon Sinopidis, Elpis Mantadakis, Katerina Kambouri

**Affiliations:** 1 Neonatal Intensive Care Unit, Alexandroupolis University General Hospital, 68100 Alexandroupolis, Greece; soulav9@hotmail.com (A.V.); emtsouvala@gmail.com (E.T.); david13xanthi@hotmail.com (R.M.); 2 Department of Radiology, Alexandroupolis University Hospital, Democritus University of Thrace, 68100 Alexandroupolis, Greece; sdefter@med.duth.gr (S.D.); foutzita@gmail.com (S.F.); 3 Department of Anesthesiology, Alexandroupolis University Hospital, Democritus University of Thrace, 68100 Alexandroupolis, Greece; pchlorop@med.duth.gr; 4 Department of Pediatric Surgery, Alexandroupolis University Hospital, Democritus University of Thrace, 68100 Alexandroupolis, Greece; bekiaridounadia@hotmail.com; 5 Department of Pediatric Surgery, Papageorgiou General Hospital, Aristotele University of Thessaloniki, 56403 Thessaloniki, Greece; xkaselas@auth.gr; 6 Department of Pediatric Surgery, University General Hospital of Patras, 26504 Rio, Greece; xsinopid@upatras.gr; 7 Department of Pediatrics, Alexandroupolis University Hospital, Democritus University of Thrace, 68100 Alexandroupolis, Greece; emantada@med.duth.gr

**Keywords:** lung, neonates, NICU, nursing, pediatric radiology, ultrasonography, chest X-rays, emergency

## Abstract

**Background:** Endotracheal tube (ETT) placement is crucial for neonates with respiratory failure. Ultrasonography (US) has emerged as a valuable tool to detect ETT positioning, competing with traditional methods. Nurses, being front-line caregivers, can perform basic ultrasound examinations. This study aimed to assess whether a nurse inexperienced in US could identify the correct ETT position in neonates after a brief ultrasound training. **Methods:** This study included intubated neonates hospitalized in a NICU with a postmenstrual age of under 45 weeks. A NICU nurse, following a short ultrasound training, measured the distance of the ETT tip to the right pulmonary artery and aortic arch. Chest X-rays (CXRs) confirmed the ETT position. The neonates’ ages, genders, weights, and examination times were recorded. **Results:** This study involved 67 neonates, including 40 (59.7%) males, with 39 (58.2%) weighing below 1500 g. The median time for correct ETT placement confirmation by CXR was 12.6 min, while US-assisted ETT recognition took 6 min initially and 5.1 min at the end of the training, which was a significant difference. No major differences were found in US distance based on the neonate’s weight and age. Gender marginally influenced US distance (β = −0.089, *p* = 0.056). **Conclusions:** The NICU nurse responded well to ultrasound training, showing results comparable with CXR. Further studies with more patients and additional studied factors are needed to fully assess US’s reliability in determining ETT positioning.

## 1. Introduction

Endotracheal intubation is a life-saving procedure, both for neonatal intensive care unit (NICU) patients with respiratory failure and in emergencies such as neonatal resuscitation in the delivery room [1]. Urgent intubation in neonates is particularly challenging, requiring a high degree of expertise, especially in preterm neonates with short and narrow airways. Misplacement of the endotracheal tube (ETT) is very common, and ensuring correct placement is essential. If the ETT is placed too high, it may lead to unintentional extubation. The incidence of esophageal intubation has been reported to be 21.4% [2], though it is typically recognized immediately. Furthermore, misplacement of the ETT in cases of respiratory distress syndrome may result in inadequate or ineffective surfactant administration [3,4].

Currently, there is no consensus on the most effective method for assessing ETT position in neonates. The ideal method should be rapid, non-invasive, reliable, radiation-free, and easy to learn and apply. The simplest, yet unfortunately inaccurate, approach involves assessing clinical signs such as a rapid improvement in heart rate and oxygen saturation, the presence or absence of thoracic contractions, symmetrical bilateral breathing sounds, and condensation in the ETT [5]. Chest X-ray (CXR) is recognized as the “gold standard” for determining the ETT tip position, but it has several drawbacks. CXR can lead to significant cumulative radiation exposure, is often delayed, requires frequent patient manipulation, and carries the risk of ETT migration.

In recent years, ultrasonography (US) has shown promise not only for diagnosing and monitoring various neonatal conditions—such as transient tachypnea, respiratory distress syndrome, pneumonia, pneumothorax, atelectasis [6,7], and bronchopulmonary dysplasia [8]—but also for instrument monitoring and positioning in clinical practice without the use of radiation [9]. US is emerging as a promising radiation-free adjunct [10], and it may be preferable for rapid, real-time assessment of ETT tip position. Several reports suggest that the relative positioning of the ETT tip in relation to nearby vascular landmarks (e.g., the proximal aortic arch or the right pulmonary artery) allows for the determination of ETT location with an accuracy comparable to that of a CXR [7,11].

Nurses are a vital resource for ensuring successful patient care and positive medical outcomes in emergency departments and intensive care units. The delivery of healthcare services relies on a cohesive team of physicians and nurses. Due to the high volume of rapidly deteriorating patients in emergency departments and intensive care units, combined with physician shortages, nurses are increasingly taking on roles traditionally performed by physicians, including US-guided venipuncture, fracture detection, volume assessment, focused ultrasound in trauma patients, soft tissue foreign body detection, and ejection fraction measurements [12,13]. However, to the best of our knowledge, there are no studies evaluating the ability of nurses to use US for confirming ETT placement. This study aimed to determine whether a NICU nurse without prior US experience can effectively identify the tip of the ETT using US following a brief training program.

## 2. Materials and Methods

### 2.1. Sample and Variables Analyzed

This study was conducted in the neonatal intensive care unit (NICU) of a tertiary care center in Northern Greece from December 2020 to September 2022. This study was approved by the hospital’s ethics committee and the NICU director (40491/16-11-2020). A NICU nurse (9 years’ clinical experience) took part in a training program as a researcher (as part of her doctoral thesis; she is also the first author of the present study), supervised by a pediatric surgeon and trained in lung ultrasound (LUS) by a pediatric radiologist. This program consisted of theoretical and practical interventions which initially lasted 3 months. The first part of the training protocol was described in detail in a previous publication by the same team [14]. In brief, during the first month, there was only theoretical training and study of relevant textbooks. In the second month, practical identification of five thoracic structures in neonates using US was conducted. After evaluating the nurse’s skills and providing new practical instructions, the final month focused on the identification of the same structures and a comparison of successful identification rates between the second and third months of training [14]. A GE Vivid T8 ultrasound machine (8 MHz curved array and an 8–18 MHz linear array transducer with a 25 mm footprint and hockey stick: L8-18i-D, GE Medical Systems Co., Ltd., Wuxi, China) was used. After the three months’ initial training, and once the nurse had become familiar with the identification of thoracic structures using US, a second training period followed. During this period (the exact timeframe cannot be determined as it depended on the availability of intubated neonates in the NICU), the nurse was trained to identify the tip of the ETT in properly intubated neonates, initially with the assistance of the pediatric radiologist (the aim was to complete at least 50 US identifications of ETT tips with the assistance of the pediatric radiologist. During the last ten USs, the radiologist acted as an observer of the procedure) and later without it (during this period, the nurse was required to independently identify and record at least fifty cases where she would recognize ETT tips). The total period of ETT tip identification, with and without assistance, lasted approximately 1.5 years.

Neonates who were intubated at a postmenstrual age < 45 weeks were included. Each eligible infant was enrolled one or more times in different intubations during the study period. Although some of the measurements involved the same patient, they were taken at different time-points. A CXR was taken in each case after intubation. Neonates with incomplete medical records and congenital anomalies, such as esophageal, tracheal, craniofacial, and cardiac anomalies, and those with generalized edema were excluded. Neonates with a body weight < 500 g or gestational age of <23 weeks were also excluded.

**Figure 1:**
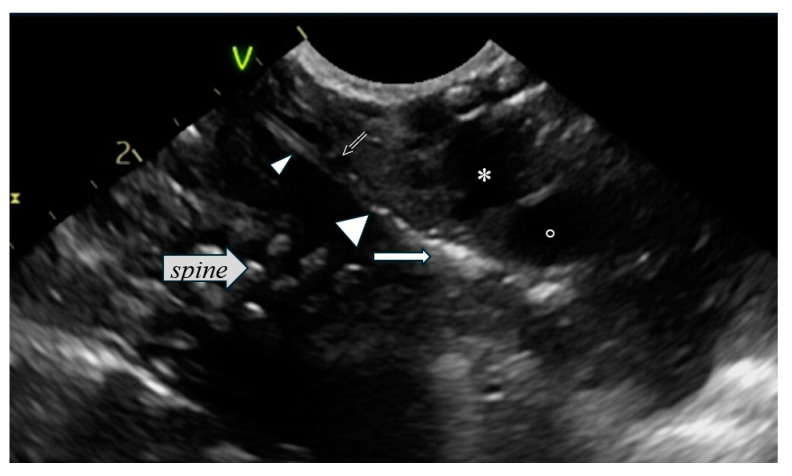
Mid-para-sagittal US view of substernal chest showing an ETT in a good position (open arrow). Note the double line of the ETT (small arrowhead) and the endotracheal “free” air (big arrowhead) resulting in a triangle hyperechoic area (white arrow), which represents the area of the carina. (*): aortic arch; (⸰): right pulmonary artery (with axial orientation in this US view).

**Figure 2:**
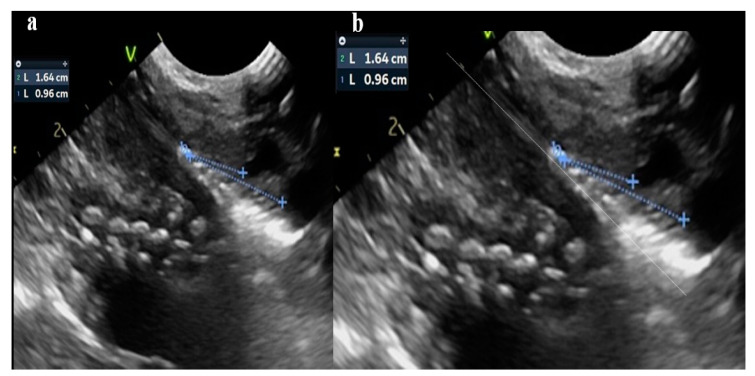
(**a**) Mid-para-sagittal US view of substernal chest showing an ETT in a good position. (**b**) The distance between the ETT tip and the aortic arch at the level of the right brachiocephalic artery (0.96 cm) [*note the “hub” on the aortic wall*] and right pulmonary artery (1.64 cm) was estimated with an angle of 15° to 20° degrees between the trachea and cephalocaudal axis (line with dots). This angulation could be the reason for the possible differentiation with radiographic measurements.

Postmenstrual age at the time of intubation, gender, body weight, and mode of delivery were prospectively documented. All intubations were performed by experienced neonatologists. The neonates were stratified into three groups. In group A (*Ν* = 67), the distance of the catheter tip from the carina was measured by CXR. In groups B (*Ν* = 52) and C (*Ν* = 57), the distance of the catheter tip from the aortic arch and the pulmonary artery was measured by US (Figure 1 and Figure 2).

All infants (*N* = 67) underwent CXR; 52 were examined by US with the guidance of an expert radiologist and 57 infants were examined by US without the radiologist’s guidance. The neonates excluded from either the first or the second US group were those whose general condition at that time was particularly critical, making it impossible to allow for US examination by the trainee. The US time required was recorded, initially during the training period with the guidance of an experienced pediatric radiologist (group B) and then without their guidance with video recording of the measurements and confirmation by the same radiologist (group C). The time required for performing the CXR was compared with the time required for US in groups B and C. Finally, we evaluated whether the ETT tip distance from the aortic arch and the pulmonary artery correlated with the weight, age, and gender of the respective neonate. The nurse’s training was considered successful firstly when the radiologist confirmed the correct identification of the ETT tip in her recorded measurements in almost 50 recordings and finally when the nurse improved the duration of clear identification. When a neonate is intubated in the NICU, the parents‘ consent is obtained by the nursing and medical staff for all procedures that will follow, as these patients require special interventions (repeated intubations, placement of central venous lines, umbilical vessels, thoracic US, repeated CXRs, and recording measurements exclusively via US). From the very beginning of intubation, parents are informed that all these procedures will be carried out as routine practice in the unit. At the same time, they are informed that some of the necessary procedures that will be performed without personal data or photographs of the newborn may be used for scientific purposes.

### 2.2. Statistics

Descriptive and inferential statistics were performed. The normality of data was checked with the Shapiro–Wilk test. The Wilcoxon signed-rank test was used to compare US time duration results before and after US training. In all other cases, parametric tests were applied (independent-samples *t*-test, Pearson correlation). Normative data for US and CXR findings for ETT tip were produced. Neonates’ weight and age on examination day were categorized according to their quartiles. A linear regression model for US distance (from the pulmonary artery) was produced. Independent variables with a *p* > 0.20 were not included in the logistic regression analysis. The level of statistical significance was set at *p* < 0.05. SPSS 2.0 was used for statistical analysis.

## 3. Results

The baseline characteristics of all enrolled infants are described in Table 1. Forty infants were males (59.7%); 39 (58.2%) were below 1500 g (BW) and 24 (35.8%) were below 28 wk.

The median duration of US was 6.00 min at the beginning of training compared with 5.1 min at the end of training, which was a significant difference (*p* < 0.001) (Table 2).

Differences between US and CXR duration were statistically significant: 6.00 (5.30–6.20) (at the beginning of training) vs. 12.60 (12.00–13.5), respectively, *p* < 0.001, and 5.10 (4.90–5.20) (at the end of training) vs. 12.60 (12.00–13.5), respectively, *p* < 0.001 (Table 3).

The distance between an optimally placed ETT tip and the carina determined by CXR was significantly and negatively **cor**related to the distance between an optimally placed ETT tip and the aortic arch determined by US (r = −0.299, *p* = 0.016) and positively correlated to the distance between an optimally placed ETT tip and the pulmonary artery (r = 0.563, *p* < 0.001) (Table 4).

US (pulmonary) distance and CXR (carina) reached higher values as age and weight increased. Regarding weight, US (pulmonary) distance (mm) increased from 14.9 (2.1) (weight ≤ 1035 g) to 15.8 (1.2) (weight ≥ 2480 g), while CXR distance (carina) (mm) increased from 11.09 (1.76) (weight ≤ 1035 g) to 11.84 (1.52) (weight ≥ 2480 g). Regarding age, US (pulmonary) distance (mm) increased from 15.0 (1.8) (age ≤ 28.57 wk) to 15.6 (1.1) (age ≥ 36.58 wk), while CXR (carina) distance (mm) increased from 11.00 (1.91) (age ≤ 28.57 wk) to 11.73 (1.61) (age ≥ 36.58 wk). On the contrary, US (aortic) distance reached lower values as age and weight increased (Table 5).

No significant difference was noted in the US distance between an optimally placed ETT tip and the pulmonary artery according to weight or age (*p* = 0.206 and *p* = 0.552, respectively) (Table 6 and Table 7).

Male infants had a lower US pulmonary distance compared with females (14.8 ± 2.0 vs. 15.6 ± 1.6, t (65) = 1 × 8.4, *p* = 0.070), while regarding US aortic distance, no substantial difference was noted between males and females (10.0 ± 1.3 vs. 9.8 ± 1.4, *p* = 0.522). No significant correlation was observed between weight and US pulmonary distance (r = 0.151, *p* = 0.222), and the same was true between age and US pulmonary distance (r = 0.165, *p* = 0.182). When gender and weight were entered into the US (pulmonary) prediction model, gender marginally correlated to US distance (β = −0.089, *p* = 0.056) (Table 8). According to this model, the mean US (pulmonary artery) distance could be predicted as follows for male infants: US distance = 15.023 − 0.892 × 1 = 15.023 − 0.892 = 14.131 (mm). Meanwhile, for females, it was equal to the constant (i.e., 15.023; gender was treated as a dummy variable, 1 coding for males and 0 for females).

## 4. Discussion

In emergency and critical care departments, nurses are an invaluable resource for providing effective patient care and achieving positive medical outcomes. Delivering health services requires full collaboration between doctors and nurses. Due to the rapid deterioration of patients in emergency and intensive care units, along with physician shortages, nurses have increasingly been taking on more tasks and responsibilities [15]. US technology has proven to be an important tool that enables healthcare professionals to provide faster and more accurate treatment. In recent years, nurses have successfully performed various procedures using US, improving the quality of care. Some procedures mentioned in the literature include US-guided vascular access, fracture diagnosis, bladder volume assessment, soft tissue foreign body detection, ejection fraction measurements, and focused sonography in trauma patients [12,16,17,18,19,20,21,22,23]. To the best of our knowledge, no studies have been conducted to assess nurses’ abilities to use US for confirming ETT placement. This study aimed to evaluate how effectively a nurse in the NICU, with no prior ultrasound experience, can identify the tip of the ETT in emergencies using US after receiving targeted training by an experienced radiologist.

In the present study, the median US duration was 6.00 min at the beginning of training and 5.1 min at the end of training, which was a significant difference. Notably, even during the initial phase of the investigation, the time required for the US was shorter than that required for CXR, which had a median duration of 12.6 min. This indicates that US is a quick and efficient examination method. Other studies have compared the time required to perform a CXR and an US for this purpose [7,24]. For example, Singh et al. evaluated the time required to obtain a CXR with that of conducting a pulmonary US. The mean (SD) time from intubation to completing an US examination was shorter than the time needed to process and deliver CXR films to the clinician. The time required for a radiograph varies depending on factors such as the setup, the availability of bedside machines and operators, and the time required to process and deliver the CXR results. On the contrary, point-of-care US and the availability of clinicians at the bedside save needless delays in confirming the ETT tip position. However, point-of-care US requires the existence of an ultrasound machine in the NICU [25]. Zaytseva et al. showed that lung US could be completed faster than radiography, with a mean duration of 19.3 min compared to 47 min, and without exposure to ionizing radiation [7]. When comparing the time reported by Zaytseva et al. to the present study, one may find our time duration surprisingly shorter. However, this discrepancy can likely be explained by the fact that in the present study, the nurse measured only the time required to confirm the correct position of the ETT tip, rather than the entire procedure starting from intubation. On the contrary, other studies such as Dennington et al. found that each lung US study took less than 5 min to complete and showed no evidence of clinical deterioration [26].

In our study, somatometric and demographic characteristics such as weight, age, and also gender were measured to determine whether the correct position of the ETT depends on these parameters. We observed that US (aortic) distance was lower in neonates weighing > 2480 g compared to those weighing < 1035 g, with values ranging from 0.94 to 1.02 cm. Regarding age, the US (aortic) distance steadily decreased as age increased. On the contrary, US (pulmonary) distance and CXR (carina) reached higher values as age and weight increased, and these metrics were positively correlated. These findings are consistent with published observations. Zaytseva et al. found that an ETT tip positioned 0.5 to 1.0 cm from the aortic arch indicates that the ETT is not “too deep” [7]. Chowdhry et al. also used US to measure the distance from the point of the maximum curvature of the aortic arch to the ETT tip, establishing a minimum distance of 1 cm to define a “not too deeply placed ETT” [27]. Similarly, Slovis and Poland demonstrated a strong association between the distance of the ETT tip to the carina on CXR and that of the ETT tip to the aortic arch on US [28]. Sethi et al. found that in 48 out of 53 appropriately inserted ETTs, the distance between the ETT tip and the aortic arch ranged from 0.5 to 1 cm [29]. On the contrary, Singh et al. observed that the ideal distance between the ETT tip and the aortic arch increased with weight and gestation [25]. Uygur et al. found that the mean (SD) distance between the ETT tip and the aortic arch was 0.78 (0.21) cm in newborns weighing < 1500 g and 1.04 (0.32) cm in infants weighing ≥ 1500 g when measured by US [30]. Also, Singh et al. discovered that the ideal distance between the ETT tip and the aortic arch increases with weight and gestation [25].

Our findings align with those of Dennington et al. and Najib et al. who assessed the distance between the ETT tip and an anatomical equivalent of the carina, i.e., the superior section of the right pulmonary artery, and discovered a strong correlation with CXR [11,26]. In the present study, a positive correlation was also observed between the distances measured by US relative to the pulmonary artery and the distances measured by CXR. It was further observed that these US measurements were more consistent than those of the CXR, which currently remains the gold-standard diagnostic technique for determining the correct ETT position. In contrast, US measurements relative to the aortic arch showed a negative correlation compared to CXR. However, to determine whether measuring the distance from the pulmonary artery is more reliable than measuring that from the aortic arch, further studies with multiple measurements by different researchers are needed.

No significant difference was found in the US-measured distance between an optimally placed ETT tip and the pulmonary artery concerning weight or age (*p* = 0.206 and *p* = 0.552, respectively). Weight may not be easily accessible shortly after birth or during emergencies, and it may be unreliable in newborns who are edematous or dehydrated [25]. However, when gender and weight were entered in the US (pulmonary) prediction model of the present study, gender was found to be marginally correlated to US distance, suggesting it could potentially serve as an indicator for predicting the correct distance of the ETT from the pulmonary artery. In the literature, other anthropomorphic measurements besides weight and age have been examined by different studies to predict the correct ETT placement. For instance, Zaytseva et al. found that derivatives of sternal length and shoulder–umbilical length may be beneficial, although these have not been completely confirmed and are not widely used [7]. The “nasal-tragus distance +1” method is considered a good predictor of the distance from the carina to the lip when selecting the ETT position [31]. However, some studies have found that this approach can result in excessive insertion depth for neonates weighing > 2.5 kg [32]. Uygur et al. refer to several methods which have been suggested for predicting ETT length in neonatal intubation, including Tochen’s formula, orohelical length, nasal tragus length (NTL), and vocal cord guidance [30]. Shukla et al. used sternal length to estimate insertion depth and claimed that, in rare situations of facial deformity, sternal length assessment could also be a valid approach for newborn intubation. In clinical practice, insertional length (IL) is often predicted using a variety of anthropometric parameters [33]. In a study by Singh et al., IL had a substantial correlation with anthropometric variables, including weight, crown heel length (CHL), NTL, occipito-frontal circumference (OFC), and sternal length [25]. Tatwavedi et al. similarly found a correlation between weight and insertional length [34]. The consistency between Tatwavedi and Singh’s findings may be due to similar populations in both studies, while differences from other studies may be due to racial variations in tracheal size [25,34,35,36].

While this study demonstrated that a nurse can be effectively trained to determine ETT placement by US, it has several limitations. This research study was conducted at a single institution with a relatively small sample size, and only one nurse performed all US examinations. Future studies should involve more neonates and multiple practitioners from many institutions to validate training methods and compare individual competencies. Additionally, while this study aimed to record ETT positions relative to the neonates’ age, weight, and gender, factors such as lip movement may have affected the measurements’ accuracy. Also, US is an operator-dependent procedure, requiring specialized training before data can reliably affect clinical management. Finally, although repeated measurements on the same neonate could introduce some bias, it should be noted that the number of patients who were measured for a second time in this study was only 12. Moreover, as previously noted, they were measured at different periods with different anthropometric characteristics, i.e., the measurements involved distinctive intubations that were assigned to a different US group.

Overall, this study showed that a NICU nurse, even without prior US experience, can accurately identify correct tracheal tube placement in neonates during emergencies following brief US training. US enabled quicker detection of the ETT tip than CXR, and the distance between the optimally placed ETT tip and carina on CXR corresponded with the distance to the pulmonary artery observed by US. However, further research with more patients, multiple researchers, and additional parameters is required to enhance accuracy and to confirm these findings across diverse clinical settings.

## Figures and Tables

**Table 1:** Baseline characteristics of overall study population.

Categories	*N*	%
Gender (male)	40	59.7
Birth weight (g)		
<1000	18	26.9
1000–1499	21	31.3
1500–1999	6	9.0
2000–2499	7	10.4
≥2500	15	22.4
Birth weight (g)-binary		
<1500	39	58.2
≥1500	28	41.8
PMA gestational (wk)		
<28	24	35.8
28–31	20	29.9
32–35	9	13.4
≥36	14	20.9
	Mean (SD)	Median (25th–75th perc.)
Weight (day of examination)	1729 (880.31)	1310 (1035–2480)
Age (wk) (day of examination)	32.69 (4.42)	31.86 (28.57–36.57)

SD: standard deviation.

**Table 2:** Comparative assessment of US duration at the beginning and at the end of training.

*Ν* = 67		USminΒ1	USminΒ2	*p*
Percentiles	5	4.900	4.500	
	10	4.980	4.600	
	25	5.300	4.900	
	50	6.000	5.100	<0.001
	75	6.204	5.200	
	90	7.940	5.600	
	95	9.760	6.200	
Wilcoxon Z = −6.954				

**Table 3:** Comparative assessment of US and CXR duration in minutes.

	USmin (Begining of Training) Percentiles 50th [25th–75th] *N* = 52	USminΒ (End of Training) (Percentiles 50th [25th–75th] *N* = 57	CXR min (Percentiles 50th [25th–75th] *N* = 67	*p*_1_ *	*p*_2_ **
Percentiles	6.00(5.30–6.20)	5.10 (4.90–5.20)	12.60 (12.00–13.5)	<0.001	<0.001
Wilcoxon Z	* for difference between US min begining of training and CXR min: −7.090 ** for difference between US min end of training and CXR min: −6.954				

**Table 4:** Pearson correlation between CXR distance and US distance.

*N* = 67		US Distance (ETT Tip–Aortic Arch)	CXR Distance (ETT Tip–Carina)
US distance (ETT tip–pulmonary artery)	r	0.563	0.044
	*p*	<0.001	0.723
US distance (ETT tip–aortic arch)	r		−0.299
	*p*		0.016

**Table 5:** Normative data of distance between optimally placed ETT tip and arch of aorta/pulmonary artery by US and CXR (carina) across different weight and age categories (*n* = 67).

Categories	US (Aortic) (mm)	US (Pulmonary) (mm)	CXR (Carina) (mm)
	Mean (SD) [95% CI]	Mean (SD) [95% CI]	Mean (SD) [95% CI]
Weight (g) *			
≤1035	10.0 (1.6) [9.2–10.9]	14.9 (2.1) [13.8–16.0]	11.09 (1.76) [10.19–12.00]
1036–1310	9.9 (1.7) [9.1–10.8]	14.4 (2.1) [13.4–15.5]	11.27 (1.54) [10.48–12.06]
1311–2480	10.2 (1.2) [9.6–10.8]	15.4 (1.9) [14.4–16.3]	11.60 (1.31) [10.93–12.28]
≥2480	9.4 (1.1) [8.8–10.0]	15.8 (1.2) [15.1–16.4]	11.84 (1.52) [11.03–12.66]
Age (in weeks) *			
≤28.57	10.3 (1.8) [0.94–1.12]	15.0 (1.8) [14.1–16.0]	11.00 (1.91) [10.02–11.99]
28.58–31.86	1.00 (0.14) [0.94–1.05]	14.8 (2.1) [14.0–15.7]	11.36 (1.33) [10.83–11.90]
31.87–36.57	0.96 (0.13) [0.86–1.06]	15.3 (2.4) [13.4–17.2]	12.04 (1.03) [11.25–12.84]
≥36.58	9.6 (1.1) [9.0–10.2]	15.6 (1.1) [15.0–16.1]	11.73 (1.61) [10.84–12.62]

* On examination day.

**Table 6:** US distance between an optimally placed ETT tip and the pulmonary artery according to the birth weight.

	Weight	*N*	Mean	Std. Deviation	Std. Error Mean	*p*
US distance	<1500 g	39	14.9	2.0	0.3	0.206
	≥1500 g	28	15.5	1.6	0.3	

**Table 7:** US distance between an optimally placed ETT tip and the pulmonary artery according to age.

	Age	*N*	Mean	Std. Deviation	Std. Error Mean	*p*
US distance	<32 w	44	15.0	2.0	0.3	0.552
	≥32 w	23	15.3	1.6	0.3	

**Table 8:** Linear regression model for US (pulmonary artery) distance.

R^2^ = 0.078	Unstandardized Coefficients		*p*	95.0% Confidence Interval for B	
	B	Std. Error		Lower Bound	Upper Bound
(Constant)	15.023	0.553	<0.001	13.920	16.130
Gender (male = 1)	−0.892	0.458	0.056	−1.638	0.015
Weight	<0.001	<0.001	0.168	<0.001	<0.001

Dependent variable: US (pulmonary artery) distance.

## Data Availability

The database of this study can be accessed upon request at the address kampouri@med.duth.gr.
